# Subnanometre enzyme mechanics probed by single-molecule force spectroscopy

**DOI:** 10.1038/ncomms10848

**Published:** 2016-02-24

**Authors:** Benjamin Pelz, Gabriel Žoldák, Fabian Zeller, Martin Zacharias, Matthias Rief

**Affiliations:** 1 Physik Department E22, Technische Universität München, James Franck Strasse 1, 85748 Garching, Germany; 2 Physik Department T38, Technische Universität München, 85748 Garching, Germany; 3 Munich Center for Integrated Protein Science, 81377 München, Germany

## Abstract

Enzymes are molecular machines that bind substrates specifically, provide an adequate chemical environment for catalysis and exchange products rapidly, to ensure fast turnover rates. Direct information about the energetics that drive conformational changes is difficult to obtain. We used subnanometre single-molecule force spectroscopy to study the energetic drive of substrate-dependent lid closing in the enzyme adenylate kinase. Here we show that in the presence of the bisubstrate inhibitor diadenosine pentaphosphate (AP5A), closing and opening of both lids is cooperative and tightly coupled to inhibitor binding. Surprisingly, binding of the substrates ADP and ATP exhibits a much smaller energetic drive towards the fully closed state. Instead, we observe a new dominant energetic minimum with both lids half closed. Our results, combining experiment and molecular dynamics simulations, give detailed mechanical insights into how an enzyme can cope with the seemingly contradictory requirements of rapid substrate exchange and tight closing, to ensure efficient catalysis.

## Abstract (web summary)

Adenylate kinase catalyses the interconversion of adenosine phosphates, and plays a crucial role in maintaining cellular energy homeostasis. Here, the authors use single molecule optical tweezers to understand how the enzyme's conformation dynamics modulates catalysis.

## Graphical abstract

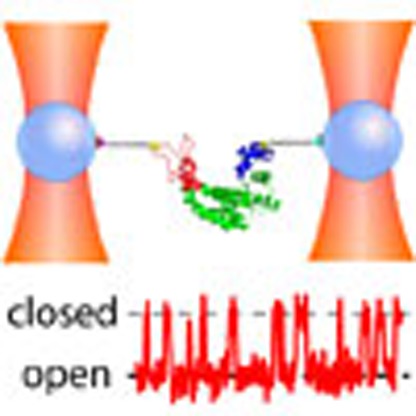

Enzymes are machines that catalyse biochemical reactions in a highly dynamic process often involving large conformational changes. How conformational changes are coupled to catalytic turnover is still controversial^1^,^2^. Experimental techniques employed to answer those important questions are often limited and, although we have high-resolution crystal structures of selected states along the reaction pathways, capturing reaction dynamics and structure at the same time is difficult. As conformational changes are transitions in an energy landscape involving interatomic distance changes, enzymes may be viewed as molecular machines^3^. Single-molecule mechanical methods such as atomic force microscopy or optical tweezers have opened new ways for studying conformational transitions of molecular motors and in protein and nucleic acid folding^4^,^5^,^6^,^7^,^8^,^9^. In most enzymes, however, conformational motions associated with catalysis are generally very small (<1 nm), involving only few residues, and hence have so far not been directly amenable to those techniques. In this study, we have employed a high-resolution optical tweezers setup to study the mechanical coupling of substrate binding and lid closing in the enzyme adenylate kinase (AdK).

AdK catalyses the reversible conversion of ATP and AMP to two ADPs and thereby plays an important role for the energy balance of the cell. Adk consists of three domains. The ATP- and AMP-lid are responsible for binding of substrates and the CORE domain governs the overall stability of the enzyme^10^,^11^. During its catalytic cycle, AdK undergoes a large conformational change as both the ATP- and AMP-lid close over the CORE domain, thereby reducing unproductive active site fluctuations and minimizing non-productive hydrolysis^12^,^13^,^14^. Opening of AdK and subsequent product release have been suggested as the rate-limiting step for enzymatic turnover^1^,^15^,^16^. A variety of experiments and simulations have indicated that the enzyme samples closed-like states even in the absence of substrates^16^,^17^,^18^,^19^. Bisubstrate analogues consisting of two adenosine groups connected by a chain of phosphates, such as AP5A, have been proven strong inhibitors of AdK and bind with nanomolar affinity^20^,^21^. AP5A is a multi-substrate inhibitor, which induces closing of the enzyme and keeps the lids firmly shut as has been shown by multiple crystal structures^1^,^14^. Therefore, the AP5A-bound state has served as a model for the fully closed (fc) state of AdK. Although crystal structures convey a picture of a tightly closed substrate-bound conformation, nuclear magnetic resonance (NMR) studies indicate much more dynamics^1^. In this study, we used single-molecule optical tweezers to directly measure the substrate-dependent forces that drive AdK into a closed conformation.

## Results

### Conformational dynamics in the presence of an inhibitor

**Figure 1:**
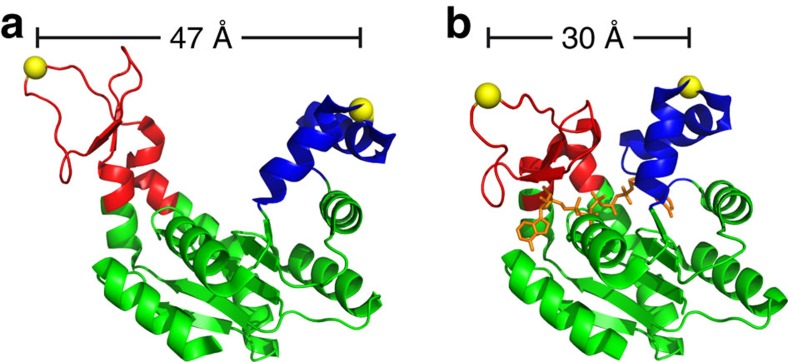
Structure of AdK. The three-dimensional structure^34^ of the open (**a**) and closed (**b**) conformation of AdK from *A. aeolicus* (PDB ID Code 2RH5 and 2RGX). The CORE domain is labelled in green, and ATP and AMP lid in red and blue, respectively. The closed conformation is shown in the presence of the bisubstrate inhibitor AP5A (orange). The yellow spheres indicate the position of the inserted cysteines between position 42 and 43, and 144 and 145. The distance between the cysteines in the open and closed conformation is shown above.

**Figure 2:**
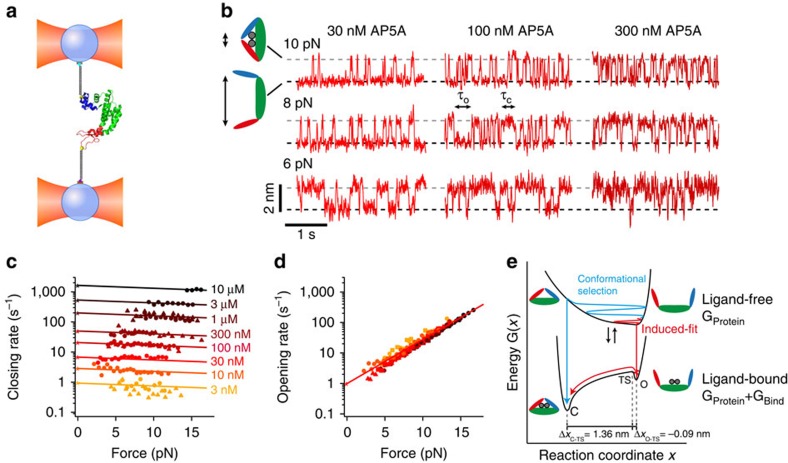
Single-molecule force experiments of AdK by optical tweezers. (**a**) Optical trap assay (for details, see Methods). (**b**) Sample traces of the closing and opening fluctuations of AdK at different Mg-AP5A concentrations and force biases. The grey and black dashed lines indicate the position of the closed (contracted) and open (extended) state, respectively (see also Supplementary Fig. 2). (**c**) Closing rate as a function of force for different Mg-AP5A concentrations. Solid lines are extrapolations of the closing rates to zero force, which are indicated by the asterisks (for details, see Methods). (**d**) Opening rate as a function of force for different Mg-AP5A concentrations (colours as in **c**) (see also Supplementary Fig. 3). The solid line is a fit extrapolating the opening rate to zero force (asterisk). (**e**) Comparison of two binding and closing models for AdK and AP5A adapted from Okazaki and Takada^45^. Binding and unbinding of the inhibitor is represented as the jump between a ligand-free and ligand-bound energy landscape. Exemplary routes from open ligand-free to closed ligand-bound form are shown for the conformational selection (blue) and induced-fit model (red). For the ligand-bound energy landscape, distances from open and closed state to the transition state from optical trap experiments are shown.

We designed a mutant of a thermophilic variant of AdK from *Aquifex aeolicus*^1^, wherein we inserted cysteine residues at positions 42 and 144 that allowed us to bind DNA handles to the enzyme and then subject the molecule to mechanical loads in an optical trap (see Figs 1 and 2a). The residues were chosen such that the distance change between the two attachment points would be maximal (*ca*. 1.7 nm) on closing and opening of the lids as judged by the crystal structure (see Fig. 1). The variant showed wild-type activity for substrate turnover in bulk assays (see Methods). In a first set of experiments, we investigated the conformational fluctuations at constant trap positions (passive mode) in the presence of the inhibitor AP5A (see Fig. 2b). The distance between the two trap centres is held constant, imposing a constant average force bias on the fluctuating beads^4^,^22^. As only the distance between the two traps is held constant, the force acting on the protein–DNA system will vary and different forces will result in different DNA extensions that will add to the measured conformational change of the protein. To remove these linker effects, we performed a DNA elasticity correction (for details, see Methods). By changing the trap distance, the applied force bias can be varied and thus shifting the equilibrium of the conformational fluctuations of the protein.

In the presence of AP5A, we observed clear two-state transitions of AdK between an extended (lower level) and a contracted state (upper level) different by *ca*. 1.6 nm in length. We identify the contracted conformation with the lid-closed conformation of AdK and the extended state with the lid-open conformation. This interpretation is supported by the finding that in the absence of an inhibitor, only the extended state is populated under load, while the contracted conformation is populated only in the presence of an inhibitor (see Supplementary Fig. 1). Our data directly show that closing and opening of both lids is cooperative, as the molecules always transition between the fully open and fc states. This interpretation is supported by a mutant where we attach our handles to only one lid (ATP lid) and the CORE domain (for details see Methods). We find conformational changes of only half the size (*ca*. 0.9 nm) albeit with identical kinetics (Supplementary Fig. 4).

The conformational transitions of AdK strictly depend on the concentration of inhibitor present (see Fig. 2c and Supplementary Fig. 5a). The closing rate rises proportionally to concentration over a large investigated range covering more than three orders of magnitude (Fig. 2c). In contrast, lid opening is independent of concentration (Fig. 2d and Supplementary Fig. 5b). The kinetics can be extrapolated to zero-force conditions (see asterisks in Fig. 2c,d). The values we find for closing (0.18 nM^−1^ s^−1^) and opening (1 s^−1^), as well as the associated equilibrium constant (3 nM) almost exactly match published values for AP5A binding^23^ and affinity^21^ measured in bulk assays for *Escherichia coli* AdK. This agreement reveals a tight coupling of inhibitor binding and lid closing in the enzyme. Apparently, the complete gain in free energy on inhibitor binding is invested to keep the lids firmly shut.

Interestingly, the force dependence of the rates differs dramatically for lid closing as compared with opening. Although lid closing exhibits only a very weak dependence on force (shallow slopes in Fig. 2c), lid-opening rates couple much more strongly and change almost two orders of magnitude over the force range investigated (Fig. 2d). The independence of the closing rate on force provides direct evidence for an induced-fit mechanism of inhibitor binding. Induced-fit describes a binding mechanism where substrate binds to an open state and alters the energy landscape such that the protein is strongly driven into the closed state (see Fig. 2e red pathway). An alternative mechanism would involve a conformational selection where the protein fluctuates between open and closed conformations in the unbound state. Here, substrate recognizes the (rare) closed conformations in this ensemble, binds and locks the protein in this conformation (Fig. 2e blue pathway). As closing rates in our measurements are force independent, a conformational-selection mechanism can be ruled out, because force would reduce the probability for finding a closed conformation in the unbound state and hence also reduce binding and closing rates.

In a conventional view, force-dependent opening rates and force-independent closing rates indicate a transition state position close to the open state (see Fig. 2e bottom) further supporting the induced-fit mechanism. It is important to note that an induced-fit mechanism is well consistent with the unbound state being flexible and able to sample closed-like conformations as has been inferred from single-molecule fluorescence experiments and molecular dynamics (MD) simulations^16^,^17^,^18^. However, we can rule out that significant inhibitor binding occurs through those closed conformations, which is consistent with the crystal structure being so tightly closed that direct binding of inhibitor into the buried binding site appears unlikely^14^.

### Nucleotide binding and conformational fluctuations

**Figure 3:**
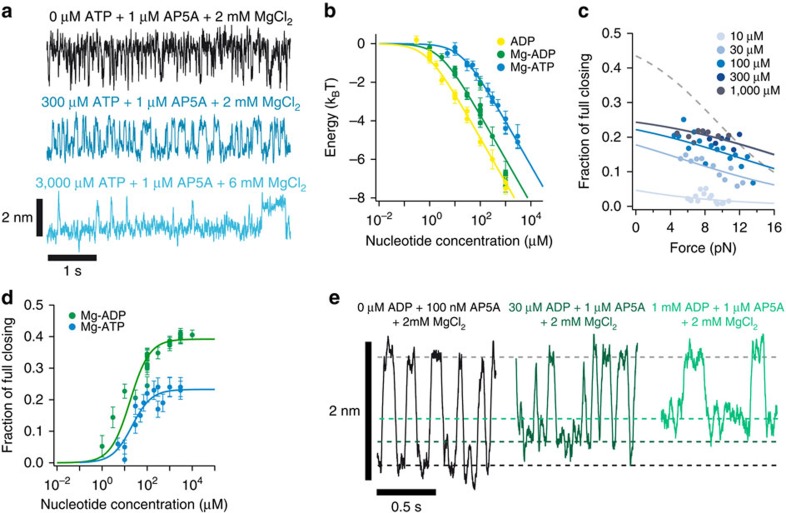
Nucleotide-induced conformational changes of AdK. (**a**) Sample traces of AP5A-induced closing and opening in the single-molecule competition assay at 1 μM AP5A with varying concentrations of ATP at a force of 10.5 pN. (**b**) Decrease in apparent binding free energy of AP5A in the single-molecule competition assay for the nucleotides ADP (yellow), Mg-ADP (green) and Mg-ATP (blue). Solid lines are fits of the binding model described in Methods (equation (2)) resulting in the following dissociation constants: 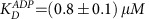, 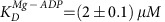 and 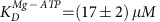. (**c**) ATP-induced fraction of full closing as a function of force for different ATP concentrations from the AP5A competition assay. Solid lines are fits to equation (7) yielding a size of the conformational change of Δ*x*=0.5 nm. The dashed line shows the force-dependent closing expected if complete ATP lid closure is assumed. (**d**) Fraction of full closing at zero force induced by nucleotides Mg-ADP (green) and Mg-ATP (blue) as a function of nucleotide concentration extracted from the AP5A competition experiments. Solid lines are fits to the data assuming a binding model (equation (12)) with an affinity of the nucleotides to open and closed state of AdK. The resulting dissociation constants are 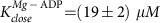, 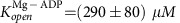 and 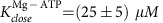 and 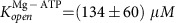. (**e**) ADP-dependent lid closing from AP5A competition experiments. The upper dashed line indicates the position of the fc AP5A-bound state and the lower three lines indicate the position of the ADP-dependent AP5A-unbound state.

Under catalytic conditions with substrates, AdK has to be much more dynamic, as the enzyme must ensure rapid turnover. In a next set of experiments, we therefore investigated the lid-closing mechanics in the presence of the substrate molecules ATP and ADP. In contrast to AP5A, we could not detect any conformational kinetics in the presence of either of the substrate molecules (Supplementary Figs 6 and 7). Given the expected fast conformational fluctuations reported in NMR and in fluorescence experiments under saturating substrate conditions^1^,^12^,^16^, we suspected those fluctuations to be buried in the noise in our experiments. To assess the magnitude of the conformational transition with substrate, we therefore designed a competition experiment with fixed concentrations of AP5A and varying concentrations of substrate molecules (see Fig. 3a). This assay allows us to monitor the full conformational transition induced by AP5A and, at the same time, follow any changes induced by substrates.

With increasing ATP concentrations (Fig. 3a top to bottom), the open dwell times increase significantly, whereas the closed dwells remain unaffected. Apparently, ATP binds to the open state and blocks rebinding of AP5A. As an example, the open dwells increase their lifetime from 5 ms (0 μM ATP, upper trace in Fig. 3a) to about 0.5 s at 3,000 μM (lowest trace in Fig. 3a). This means that during 99% of the open dwell time, ATP is bound to at least one binding site, therefore blocking the rebinding of AP5A from solution. This increase in dwell time provides a measure for the binding affinities of the various substrates to AdK (Fig. 3b and for details, see Methods). All affinities agree within error with published affinities from bulk experiments with no mechanical loads applied^21^, proving that our tethered enzyme has fully native substrate-binding properties. Further evidence that substrates bind directly to the enzyme even without AP5A present comes from experiments where we pulled AdK to forces where part of the structure unfolds (Supplementary Fig. 6). Constant velocity experiments in the presence of nucleotides (see Supplementary Fig. 6) suggest that the observed equilibrium transition is caused by the folding and unfolding of the ATP lid. This is supported by previous studies showing that the ATP and AMP lid of the mesophilic AdK from *E. coli* have a lower thermodynamic stability than the CORE domain with the ATP lid unfolding already at temperatures over 35 °C (refs 11, 24). The higher conformational stability of the hyperthermophilic variant from *A. aeolicus* we use in our study explains why ATP lid melting is negligible at room temperature and needs substantial mechanical force to be induced. We observe that in the presence of substrates this equilibrium unfolding/folding transition is shifted to higher values demonstrating that substrate has bound and stabilized the protein structure. From these experiments we can independently extract dissociation constants from substrate binding, which lie in a very similar range as the ones obtained from the competition assay (for details, see Methods and Supplementary Fig. 8).

In contrast to AP5A (see above), the application of load apparently does not affect the binding of substrates to AdK as can be seen from the similarity of solution affinities with the ones we obtain at a pulling force of 10 pN. This provides evidence that substrate binding is not coupled to significant closing of the enzyme. Our competition assay also yields a direct comparison of the degree of lid closing between the substrate-bound and inhibitor-bound states. Even though our temporal resolution does not allow us to capture the individual conformational fluctuations induced by the rapid substrate binding/unbinding events, we can make a clear statement about the average degree of closing. Again, in the case of 3,000 μM ATP (see lower trace in Fig. 3a), the apparent closing distance between open and closed dwells induced by AP5A binding is almost identical to the one measured without ATP. This means that even though AdK carries ATP during 99% of the time in the open dwell, on average, the enzyme is nearly open. If ATP binding induced full closing of the ATP lid, the apparent position of the open dwell level would have to be half its original value.

A closer analysis of the substrate-induced shift of the lower dwell levels is shown in Fig. 3c. For ATP, we find a substrate-dependent shift of the lower level (fraction of full closing) of *ca*. 0.2 as compared with the full closing distance. Apparently, the lid closes, but much less than full closing of one lid (0.5), let alone both lids (1.0). Two possible explanations outlined in Supplementary Fig. 10 could explain this result: (1) ATP binding occurs to a conformation with an only partially closed lid (pc) or (2) substrate binds to the open state with full affinity but drives AdK only weakly into a fc conformation resulting in a rapid equilibrium between fc and the open (o) conformation. Both of these explanations would support a conclusion that ATP binding does not strongly drive AdK into an fc ATP-lid state.

Analysis of the force dependence of the fraction of full closing (Fig. 3c) provides evidence for explanation (1) and hence the existence of a free-energy minimum in the AdK energy landscape at a pc position. We find that force only weakly affects the fraction of full closing as can be seen from the shallow slopes of the data points at all ATP concentrations. A fit to the data is consistent with a pc state (solid line fits) but not with an fc lid (dashed line; for a detailed model, see Methods). We cannot exclude transient population of the fc state; however, the major population will be the pc state. The concentration-dependent degree of ATP-induced lid closing extrapolated to zero force is shown in Fig. 3d (blue).

Similarly, in the presence of ADP, which binds to both lids, we also do not observe full closing of the enzyme. Even at the highest concentrations of ADP (1–10 mM) (see right trace in Fig. 3e), the ADP-bound level only approaches half of the fc state (Fig. 3d).

### Phosphate linker length of inhibitor affects closing

**Figure 4:**
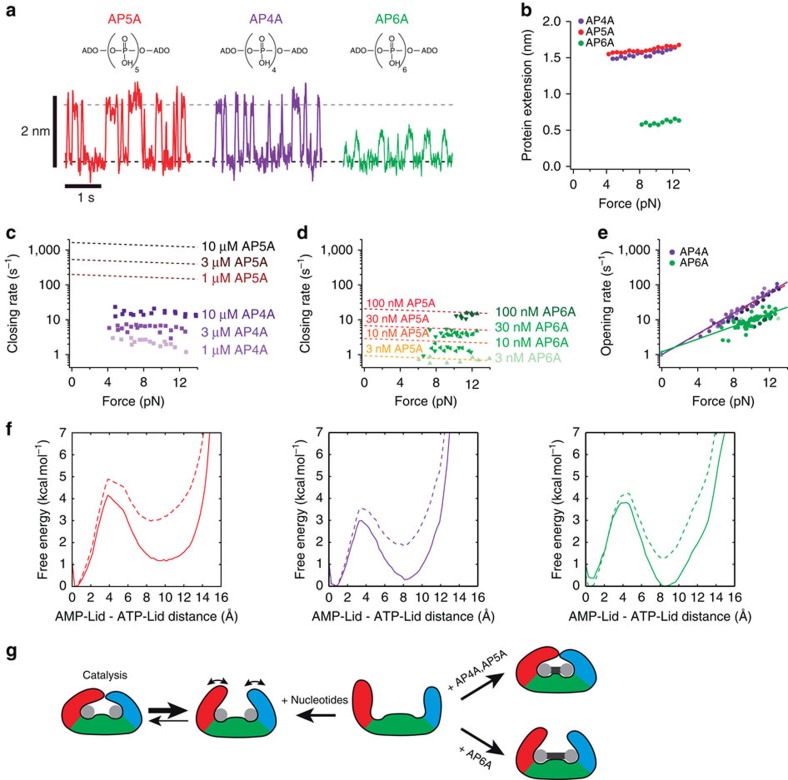
Effect of the phosphate linker length between the adenosine moieties. (**a**) Equilibrium traces of the closing and opening of AdK in the presence of AP5A (left panel), AP4A (middle panel) and AP6A (right panel) at a force of 8 pN for AP5A and AP4A, and 12 pN for AP6A. The dashed lines represent the position of the fc (grey) and open (black) state of AdK. (**b**) Distance between closed and open state as a function of force in the presence of the different inhibitors AP5A (red), AP4A (purple) and AP6A (green). (**c**–**d**) Closing rate of AdK as a function of force for different AP4A (**c**) or AP6A (**d**) concentrations. It is noteworthy that the dashed lines are not fits to the AP4A and AP6A data, but fits to the rates obtained at the corresponding AP5A concentrations for comparison. (**e**) Opening rate of AdK as a function of force for different AP4A (purple) and AP6A (green) concentrations. Solid lines are fits to the closing rates extrapolated to zero force (asterisks). For comparison, a fit to the rates obtained at the corresponding AP5A concentrations is shown as a red dashed line. (**f**) Calculated free energy along the opening of AdK bound to AP5A (red), AP4A (purple) and AP6A (green) obtained from free-energy simulations without load (dashed lines) and with a subsequently added bias corresponding to an external load of 15 pN (solid lines). The free-energy curves are projections of free-energy landscapes (Supplementary Fig. 11) obtained from 2D H-REMD-US simulations in which the arrangements of AMP lid and ATP lid were controlled independently. (**g**) Model of the coupling between the conformational fluctuations and the binding of inhibitors or nucleotides to AdK. In the presence of nucleotides, a pc state is stably populated under force; however, AdK is still able to close fully especially in the absence of force. The binding of AP4A and AP5A induce the fc conformation of AdK, whereas the binding of AP6A stabilizes a pc state.

How does the strong energetic drive towards the closed state of the enzyme in the presence of AP5A fit together with the population of a pc state and only a weak drive towards the fc state in the presence of substrates? A major difference between AP5A and two individual substrates is the rigid linkage between the adenosine moieties in AP5A, while individual substrates are free to move relative to each other. The rigid linkage in the inhibitor probably puts significant constraint on the conformational flexibility of AdK. Coupling of the relative distance between the substrate-binding sites to the degree of lid closing could explain why the inhibitor locks the enzyme into the closed state: if only the closed conformation offers a binding site distance that matches the length of the phosphate linker, AP5A can only bind tightly to the closed state. We tested this hypothesis by measuring lid-closing distances with the inhibitors AP4A and AP6A, where the two adenosine moieties are linked with four or six phosphate groups, respectively (see Fig. 4a), thus altering their relative distance.

We found that AP4A induces full closing of AdK indistinguishable from AP5A (see Fig. 4b). In contrast, AP6A only closes to about 0.6 nm, corresponding to a fraction of full closing of 0.4. One might argue that reduced closing due to AP6A as compared with the two other inhibitors is owed to the longer and bulkier phosphate chain that cannot be completely accommodated into an fc structure. However, the substrate-dependent closing and opening rates suggest a different explanation: we find that AP4A has a 100-fold reduced closing rate (see Fig. 4c), which is consistent with a similarly reduced affinity for AP4A measured in bulk experiments^21^. For AP6A, we measure closing/binding rates that are identical to the ones of AP5A, showing that AP6A binds with the same affinity as AP5A despite its significantly reduced closing distance (see Fig. 4d). Zero-force opening rates for AP5A, AP4A and AP6A are largely identical (see Fig. 4e). It is noteworthy that the force dependence for opening of AP6A is weaker (smaller slope), consistent with the smaller conformational change (see Methods). Our results suggest that if the distance between the two adenosine moieties is larger, full lid closing for high affinity binding is not required. Hence, under these conditions, a pc state is already an optimal configuration.

Atomistic MD free-energy simulations of AdK bound to all three inhibitors support our conclusion and provide additional structural insight (Fig. 4f and for details, see Methods). In all cases, a significant free-energy minimum at a lid closing of ∼50% was observed, consistent with our interpretation that a pc state of the enzyme is a dominant feature of its energy landscape. For AP4A and AP5A binding with an added load of 15 pN, the fc state is of lower free energy than the pc state. In contrast, under the same conditions, for AP6A the pc state is lower in free energy than the fc state. Although a precise quantitative prediction of free energies cannot be expected given the known shortcomings of MD simulations, this result is at least in qualitative agreement with the experiment and in line with the experimentally observed dominant closing fractions in the inhibitor-bound states. Analysis of the simulation trajectories offers a structural explanation (see Supplementary Fig. 11). For the longer inhibitors, especially AP6A, a simultaneous binding of the two adenosine moieties to the corresponding lid sites is possible already at partially open AdK conformations. In case of the shorter AP4A inhibitor this stable binding mode is only possible at more closed lid configurations, which also offers an explanation for the reduced closing rate of AdK when AP4A is bound.

## Discussion

The possibility of studying native-state conformational energies using mechanical single-molecule methods allows us to quantitatively determine the energetic drive of conformational changes of an enzyme induced by substrate binding. Even though structural studies have suggested firmly closed states, our results now provide an important enhancement of our understanding of AdKs conformational dynamics. The two apparently contradictory requirements of firm closing ensuring catalytic activity and flexibility to ensure rapid substrate/product exchange are met by (1) a strong drive towards a pc state with high substrate affinity still allowing for substrate exchange and (2) a much weaker drive into an fc and catalytically active structure that is only transiently visited. These results show that subnanometre force spectroscopy opens up new possibilities to directly study the energetic balance of enzyme conformations and help answering the question how these conformations couple to substrate binding.

How can our results be reconciled with reports from the literature that substrates induce full closing of AdK^1^,^12^,^25^? It is important to note that our measurements always occur under a certain amount of mechanical load. Therefore, a slight bias within the enzyme towards an fc state at zero load is well consistent with our findings. For example, Wolf-Watz *et al*.^1^ reported that under saturating substrate condition AdK rapidly fluctuates between closed and open conformations with *k*_close_=1,370 s^−1^ and *k*_open_=40 s^−1^, whereas Hanson *et al*.^16^ measure *k*_close_=400 s^−1^ and *k*_open_=160 s^−1^ for *E. coli* AdK. Both these measurements indicate the open state is populated to between 3 and 30%, consistent with our finding that despite the large substrate concentrations, the binding energy does not shift the equilibrium strongly to the closed state. Our data now explain where most of the substrate-binding free energy goes: it is used for populating a pc state. This may be important for the function of AdK as an enzyme that rapidly turns over a substrate and has to allow quick substrate exchange at the same time.

The results from the experiments with the three bisubstrate inhibitors AP4A, AP5A and AP6A can give a structural explanation for the occurrence of the pc state. Although AP4A and AP5A induce conformations where the adenosine moieties are close together, AP6A-bound AdK settles at a conformation where they are further apart (Fig. 4g right). The left branch of Fig. 4g provides a picture of how the different states might blend into the catalytic cycle of AdK: when two unlinked substrate nucleotides bind, they will rapidly settle at an energetic minimum where the lids are half-closed with little energetic drive bringing them to the fc state. This will be the main substrate-bound state of the enzyme. In this state, catalysis cannot occur, because substrates are too far apart. Transiently, the fc state will then be visited in a process driven by thermal fluctuations. It is noteworthy that the fc state may even be a high-energy state and thus not energetically favoured (that is, no favourable energy bias) at least under the forces we apply. This fc state is essential for catalysis, because water molecules need to be excluded to ensure efficient phosphate transfer^12^,^13^. Even more than a single transition to the fc state may be necessary until catalysis occurs. After catalysis has occurred, the enzyme will switch back to the half open state and eventually release products. For rapid substrate turnover, it is important that the lids be not locked by favourable energy bias in an fc state but they open rapidly to allow product–substrate exchange. We think that the pc state ensures both rapid substrate exchange and high-affinity binding through establishing contacts between substrates, core domain and lids.

Why can we not see a kinetic time constant attributable to catalysis directly in our data? A turnover rate of 25 s^−1^ (that is, 40 ms) in solution lies within the limit we should be able to resolve even in our filtered traces. However, as the fc state is only transiently populated, the lids will be only fully closed during a small fraction of those 40 ms. In this case, we are not able to pick up a signal related to catalysis in the assay. In this context, it is important to note that we only have a readout for length changes but not for chemical changes.

Moreover, in our assay, we always apply loads around 10 pN, which, in turn will speed up opening kinetics and slow down closing kinetics. If force slows transitions to the fc state also, catalysis will be slowed and may be even suppressed, offering another explanation for why we cannot observe a signal associated with catalysis. A combination of mechanical and fluorescent single-molecule measurement may, in the future, give more insight into coupling between mechanical and chemical steps.

## Methods

### Experimental procedures

The setup used was a custom-built, dual-beam, high-resolution optical trap setup as described previously^26^,^27^. Data were collected at 150 kHz and then averaged to 30 kHz before storage. Transitions between open and closed, and closed and open state were detected using Hidden Markov Model analysis on the unfiltered raw data of the difference signal of the two traps as described before^28^. The data at constant trap position shown throughout the study were low-pass filtered to 37.5 Hz. All measurements were performed in 50 mM Tris pH 7.5, 200 mM KCl, 26 U ml^−1^ glucose oxidase, 17,000 U ml^−1^ catalase, 0.65% glucose with varying concentrations of nucleotides, inhibitors, MgCl_2_ and EDTA. The MgCl_2_ or EDTA concentration was 2 mM for nucleotide and inhibitor concentration below 1 mM, and twice the nucleotide concentration for nucleotide concentrations above 2 mM. All errors given are statistical errors (1 s.d.) derived from the Levenberg–Marquardt fit algorithm.

### Protein expression and purification

The gene for AdK of *A. aeolicus* was synthesized by Mr Gene GmbH (Regensburg). The gene was cloned into pET11a using BamHI and NdeI restriction sites. Cysteine residues were inserted between positions 42 and 43, and 144 and 145 (wt-numbering) by QuikChange mutagenesis (Agilent). To observe conformational fluctuations of the ATP-lid only, a second mutant was produced with a cysteine at the amino terminus and a cysteine residue inserted between positions 144 and 145 (AdK N-144). The proteins were expressed in *E. coli* BL21(DE3) and purified by a Ni-NTA affinity column followed by gel filtration chromatography (HPLC system, Jasco Germany, GmbH) using YMC diol-120 columns (YMC Europe, GmbH) and Superdex S200 (10/300 GL, GE Healthcare). Attachment of oligonucleotides and purification optical trap assays were performed as described previously^4^,^29^, with an additional Ni-NTA affinity purification step for protein-oligonucleotide purification.

### Protein sequences

In the following, the cysteines that are used for handle attachment are shown in bold, the sequence corresponding to AdK is shown in italics and the spacers are underlined.

*AdK 42–144*. MAAKGELSG*MILVFLGPPGAGKGTQAKRLAKEKGFVHISTGDILREAVQKG**C**TPLGKKAKEYMERGELVPDDLIIALIEEVFPKHGNVIFDGFPRTVKQAEALDEMLEKKGLKVDHVLLFEVPDEVVIERLSGRRINPETGEVYHVKYNPPPPG**C**VKVIQREDDKPEVIKKRLEVYREQTAPLIEYYKKKGILRIIDASKPVE EVYRQVLEVIG*DGNGTGSKALEHHHHHH.

*AdK N-144*. MA**C**KGELSG*MILVFLGPPGAGKGTQAKRLAKEKGFVHISTGDILREAVQKGTPLGKKAKEYMERGELVPDDLIIALIEEVFPKHGNVIFDGFPRTVKQAEALDEMLEKKGLKVDHVLLFEVPDEVVIERLSGRRINPETGEVYHVKY NPPPPG**C**VKVIQREDDKPEVIKKRLEVYREQTAPLIEYYKKKGILRIIDASKPVEEVYRQVL EVIG*DGNGTGSKALEHHHHHH.

### Enzymatic activity of AdK

The enzymatic activity of the AdK mutant was tested by a coupled spectrophotometric assay^30^ (Supplementary Figs 12 and 13). The resulting Michaelis–Menten constant *K*_m_ (41±11 μM for ATP, ∼10 μM for AMP and 360±80 μM for Mg-ADP/ADP) and the catalytic rate *k*_cat_ (25±7 s^−1^) are in good agreement with the values of the wild-type protein reported by Wolf-Watz *et al*.^1^

### DNA elasticity correction

As our measurements occur at non-constant force, comparing the conformational changes of AdK under different experimental conditions requires corrections for trap and DNA compliances. We therefore corrected all traces obtained at constant trap distances for the non-Hookean elastic behaviour of the double-stranded DNA linker. Briefly, a force–extension curve of a DNA–protein construct was measured. Data, force versus time, were acquired, and from known distance and trap stiffness, force–extension curves were calculated. Next, the data were fitted by worm-like chain (WLC) model to account for the DNA tether (∼360 nm). From the now known WLC model parameters, the extension of the DNA handles can be calculated for each data point and subsequently subtracted. This ‘constant-force' transformation removes variations in the DNA linker elasticity and trap stiffness, and allows for a better comparison of the conformational changes of AdK between different experiments. All data shown are subjected to this procedure.

### Extrapolation of closing and opening rates

For determining the opening and closing rates at zero force *k*_0,*i*_ and distances to the transition state Δ*x*, the force-dependent opening and closing rates *k*_*i*_(*F*) were fitted with a Bell model^31^:


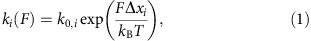


where *k*_B_ is the Boltzmann constant and *T* is the absolute temperature. The distance to the transition state is a measure for the force dependence of the respective rate. A small Δ*x* results in a small force dependence of the corresponding rate, whereas a large Δ*x* causes the opening or closing rate to be strongly dependent on force. This behaviour can be observed for the conformational change in the presence of AP5A and AP6A. Both feature a similar zero-force opening rate; however, the force dependence of the AP5A opening rate is much bigger, as the size of the conformational change and with it the distance to the transition state is larger.

Bell model fits were performed as global fits to the closing rate for the individual inhibitors, assuming the same distance to the transitions state Δ*x* for all concentrations and individual zero force closing rates *k*_0,close_. The Bell model fits to the opening rate were performed as global fits for the individual inhibitors, assuming the same distance to the transitions state Δ*x* and the same zero force opening rate *k*_0,open_.

### Equilibrium free energies

Equilibrium free energies of the conformational fluctuations of AdK in the presence of inhibitors and in the competition assay were calculated as described previously^4^.

### Affinities from competition assay

The affinity of the substrates to AdK was determined as follows. First, the binding free energy of AP5A at various AP5A concentration, Δ*G*^0^, was determined in the absence of nucleotides as described before. Second, the decrease in binding free energy due to nucleotide binding in the AP5A competition assay was determined by comparison with the AP5A-only conditions. The nucleotide concentration-dependent decrease in binding free energy can be described by


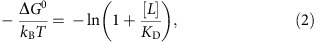


where [*L*] is the nucleotide concentration and *K*_D_ the dissociation constant of the nucleotides to AdK^32^.

### ATP lid unfolding and free energy

Stretching AdK at positions 42 and 144 shows a continuous hump-like equilibrium transition around a force of 17 pN in force extension traces (see Supplementary Fig. 6). These fast unfolding and folding transitions can be observed between 13 and 20 pN. To calculate the free energy of this transition, we integrated the force distance curves. The free energy of the whole system, including the stretching of the DNA linker, is given by the area under the force distance curve of a constant velocity experiment (for a clearer representation, Supplementary Fig. 8a shows the corresponding curves as force extension traces). To calculate the energy of the system in the unfolded state, a serial combination of DNA and polypeptide elasticity is fit to the data and the energy of the system in the unfolded state is given by the area under the fit. To reduce the influence of noise at low forces, a WLC model is also fit to the folded state. The energy of the whole system is calculated by the sum of the area under the WLC fit to the folded state up to a force of 10 pN and the area under the actual curve (orange) for forces above 10 pN. By subtracting the free energy of the system in the unfolded state (grey area) from the free energy of the whole system, we obtain the free energy of the folding/unfolding equilibrium transition (blue area).

The free energy of the folding/unfolding equilibrium transition in apo conditions is (11.7±0.7)*k*_B_*T*. The increase in free energy of this transition in presence of the nucleotides Mg-ADP and Mg-ATP is shown in Supplementary Fig. 8b.

The free energy in the presence of nucleotides can be described similar to the competition assay as


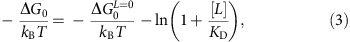


where [*L*] is the nucleotide concentration, 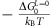 is the free energy in apo conditions and *K*_D_ the dissociation constant of the nucleotides to AdK.

### Nucleotide-induced conformational fluctuations

To determine the size of the conformational change induced by the nucleotides, the measured size of the AP5A-induced conformational fluctuations in the competition experiment is compared with the one at AP5A-only conditions. The fraction of closing induced by the nucleotides in the competition experiment is given by the difference between the size of the conformational change at AP5A-only conditions compared with the one in the presence of nucleotides in the competition experiment and normalized by the length of the conformational change in presence of AP5A only:


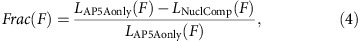


where *L*_AP5Aonly_(*F*) is the size of the full conformational change for AP5A-only experiments (in nm) and *L*_NuclComp_(*F*) is the size of the conformational change in the nucleotide competition experiment. The system can be described by the probability to find the enzyme in either the open or closed state. The force-dependent probability to find the enzyme in the closed state is given by


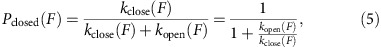


where *k*_close_(*F*) and *k*_open_(*F*) are the force-dependent closing and opening rates at a specific nucleotide concentration, respectively. Assuming the Bell model, with a zero force rate *k*_0,*i*_ and a distance to the transition state Δ*x*_*i*_, the force dependence of the rates is given by equation (1).

With equation (1), the force dependence of the probability to find AdK in the closed state can be rewritten as:


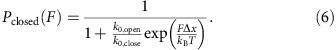


Here, *k*_0,open_ and *k*_0,close_ are the zero-force opening and closing rates, respectively, and Δ*x* is the size of the conformational change that AdK undergoes in the presence of nucleotides. To calculate the fraction of closing, equation (6) has to be extended by 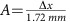



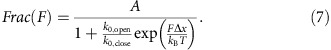


A global fit of this model to the force-dependent fraction of closing at all ATP concentration was performed. One Δ*x* for all ATP concentrations was used and an ATP concentration-dependent ratio 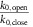 was used as well. The global fit to the data yields the size of the conformational change induced by ATP of Δ*x=*0.5 nm. A similar global fit to the force-dependent fraction of closing shown in Supplementary Fig. 9 for different Mg-ADP concentrations yields the size of the conformational change induced by Mg-ADP of Δ*x=0.7* nm. From the acquired fit parameters, the fraction of full closing at zero force can be determined (Fig. 3d).

To describe the concentration-dependent closing of AdK in presence of a ligand, the probability to find the enzyme in the closed conformation in the presence of a ligand is calculated


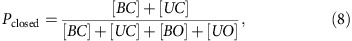


where [BC] and [UC] are the concentrations of ligand-bound and ligand-unbound closed state, respectively, and [BO] and [UO] are the concentrations of ligand-bound and ligand-unbound open state, respectively. The dissociation constants for the open *K*_open_ and closed state *K*_close_ are defined by


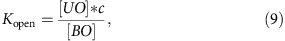


and


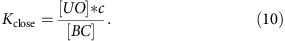


Single-molecule fluorescence experiments by Henzler-Wildman *et al*.^18^ have shown that the equilibrium between open and closed state in the absence of nucleotides lies on the open side (opening:closing rate, 6,500 s^−1^:2,000 s^−1^). Assuming a similar Δ*x* for full closing as in the AP5A case, force will shift the equilibrium further towards the open side. At our measurement forces, the concentration [UC] will be negligible compared with the other concentrations. Together with equations (9) and (10), equation (8) can be simplified to:


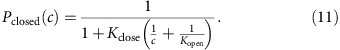


As the size of the conformational change caused by the nucleotides is known from the fits of the force-dependent closing data, the concentration-dependent fraction of closing can be written as:


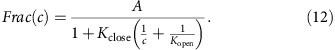


### MD simulation

All-atom MD simulations were performed on AdK bound to each of the three inhibitors AP4A, AP5A and AP6A. Two-dimensional (2D) potentials of mean force (PMFs) along the opening of the two flexible lid domains were calculated by means of Hamiltonian-replica exchange umbrella sampling (H-REMD-US) simulations. The resulting 2D free-energy landscapes were projected on a one-dimensional coordinate, resembling the experimental lid-to-lid distance. A free-energy bias corresponding to a force of 15 pN was subsequently added to the free-energy projections, to enable direct comparison with the experiments.

### Starting structures

The crystal structure of *A. aeolicus* AdK in complex with AP5A was taken from PDB:2RGX^18^. Structures of AP4A and AP6A were built manually and optimized with respect to their internal energy using Antechamber as part of Amber14 (ref. 33). Starting structures of AdK in complex with AP4A and AP6A were constructed by aligning^34^ these inhibitors over AP5A in 2RGX and subsequently removing AP5A (followed by energy minimization). Using the Leap module of Amber14, a periodic octahedral box with edge length ∼28 Å was constructed around each protein–inhibitor complex and filled with ∼9,000 water molecules. Potassium ions were added to neutralize the overall charge of the systems.

### Simulation setup

All simulations were carried out using Amber14 (ref. 33). The protein was described by the Amber ff14SB force field^35^ and the water molecules by the TIP3P model^36^. The AP4A, AP5A and AP6A inhibitors were parametrized with Antechamber and the GAFF^37^ force field. Specific parameters were used for the potassium ions^38^. The short-range cutoff radius was 9 Å. Long-range interactions were accounted for by the particle mesh Ewald method (Amber14 default parameters). Hydrogen bonds were constrained via the SHAKE^39^ algorithm. The integration time step was 2 fs. For temperature and pressure coupling, Berendsen thermostat and barostat^40^ were used with a coupling time of 1 ps.

### Equilibration

The starting structures were minimized with respect to their internal energy using the steepest decent method for 10,000 steps. Several equilibration simulations of 20 ps were performed in the NPT ensemble at a pressure of 1 bar. Initially, the temperature was successively raised to 100, 200 and 298 K, while harmonic positional restraints of a force of 50.0 kcal mol^−1^ Å^−2^ were applied to protein and inhibitor atoms with respect to the starting structures. In five additional simulations, the restraining potential force was successively reduced to 24.0, 12.0, 6.0, 2.0 and 0.2 kcal mol^−1^ Å^−2^. A final equilibration step of 40 ps was performed without restraints. The total equilibration time of each system was 2 ns.

### 2D H-REMD-US simulations

The US simulations were performed in the NVT ensemble at 298 K. The reaction coordinates used for the 2D US^41^ simulations were defined as the centre of mass distances between the C_α_-atoms of residues 33–58 and 114–123, 154–170 (AMP-lid opening) and 125–148, and 1–28 and 75–82 (ATP-lid opening), respectively. Harmonic umbrella potentials were placed along the AMP-lid coordinate between 18.0 and 30.1 Å, and along the ATP-lid coordinate between 29.0 and 31.1 Å with an intermediate spacing of 1.1 Å. To obtain fast equilibration of the US simulation setup, two different starting configurations for each umbrella window were generated. The equilibrated structures were driven along the ATP-lid coordinate and then along the AMP-lid coordinate by applying the umbrella potentials one after another with a force constant of 4 kcal mol^−1^ Å^−2^ for 100 ps. The process was then repeated in the reverse order, starting from the obtained open AdK conformation. Only during this initial process, the inhibitor atoms were restrained to their initial positions with a force of 0.025 kcal mol^−1^ Å^−2^, to prevent dissociation due to the fast initial opening motion. In this way, starting configurations generated on the basis of a completely open and a completely closed structure were provided for each umbrella window position. Subsequently, two strands of 2D US simulations with a harmonic force constant of 2 kcal mol^−1^ Å^−2^ were run in parallel, each based on one of the previously obtained sets of starting configurations. Via Hamiltonian replica exchanges^42^, neighbouring windows within and between the two strands were allowed to swap configurations. This enables rapid diffusion of the configurations along the reaction coordinate plane and between the strands as well. The exchange scheme was realized by performing 4D H-REMD-US with adequately modified exchange groups. As starting configurations originating from two configurational extremes are provided, which are allowed to rapidly exchange towards an adequate umbrella window position, equilibration is drastically sped up in comparison with a setup based on only one starting configuration per window. For the actual sampling, the umbrella windows at the positions between 29.0 and 30.1 Å, 18.0 and 21.3 Å (top left region), and between 19.0 and 22.3 Å (bottom right region, see Supplementary Fig. 11) of the reaction coordinate plane were omitted, to obtain an advantageous number of 256 parallel simulations. Replica exchanges were attempted every 1 ps with rates of successful exchanges between 0.06 and 0.29. Each umbrella window was simulated for 6 ns (12 ns per umbrella window position), totalling up to >1.5 μs of sampling time for each system. Reaction coordinate values were saved every 0.1 ps. The 2D PMFs were calculated from the sampling data using the Weighted Histogram Analysis Method^43^,^44^. The first 2 ns of sampling time were omitted in the PMF calculations, to ensure adequate equilibration of the umbrella windows.

### Free-energy projection and additional external force

To enable a direct comparison of the MD results with the experiments, the 2D free -energy landscapes obtained from the simulations (Supplementary Fig. 11) were projected on a coordinate roughly resembling the lid-to-lid distance as follows:


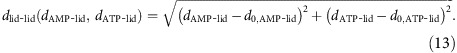


Here, *d*_AMP-lid_ and *d*_ATP-lid_ are the lid opening coordinates used in the H-REMD-US simulations, and *d*_0,AMP-lid_ and *d*_0,ATP-lid_ are the locations of the global minima in the free-energy landscapes. We hereby assumed that the lid-domain motions are mainly orthogonal. A bias corresponding to the load exerted during the experiments was subsequently added via


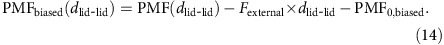


As an external force *F*_external_ 15 pN was used, representative for the loads applied during the experiments. Finally, the resulting new minima of the biased PMFs were shifted to zero.

## Additional information

**How to cite this article:** Pelz, B. *et al*. Subnanometre enzyme mechanics probed by single-molecule force spectroscopy. *Nat. Commun.* 7:10848 doi: 10.1038/ncomms10848 (2016).

## Supplementary Material

Supplementary Information Supplementary Figures 1-13 and Supplementary Reference
