# Supplementary material for: Subnanometre enzyme mechanics probed by single-molecule force spectroscopy
Source: Nat Commun. 2016 Feb 24;7:10848. doi: 10.1038/ncomms10848 (PMC4770092; doi:10.1038/ncomms10848)
Supplement: Supplementary Information — Supplementary Figures 1-13 and Supplementary Reference [file ncomms10848-s1.pdf]

Supplementary Figures

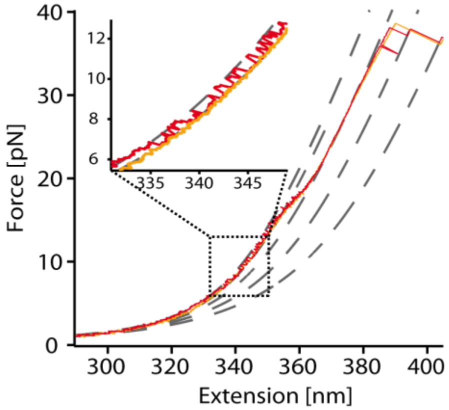

Supplementary Figure 1. Force-extension curves of AdK pulling at residue 42 and 144. The protein construct was stretched under apo conditions (orange) and in presence of 100 nM AP5A and 2mM $MgCl_2$ (red) at a constant velocity of 20 nm s^{-1} (low pass filtered to 60 Hz). The dashed lines correspond to WLC fits to the data yielding an overall contour length increase of the fully unfolded protein of 29 nm. The inset shows a magnification of the force-extension trace showing closing and opening fluctuations in the presence of AP5A. No conformational fluctuations can be observed under apo conditions.

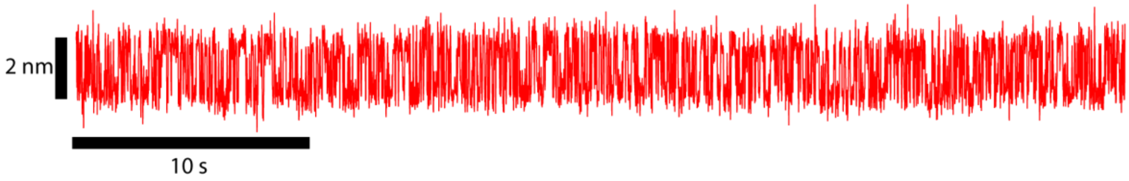

Supplementary Figure 2. Long sample trace of the closing and opening fluctuations of AdK (42-144 variant) in the presence of 100 nM AP5A and 2 mM $MgCl_2$ at a force bias of 9.5 pN for 40s.

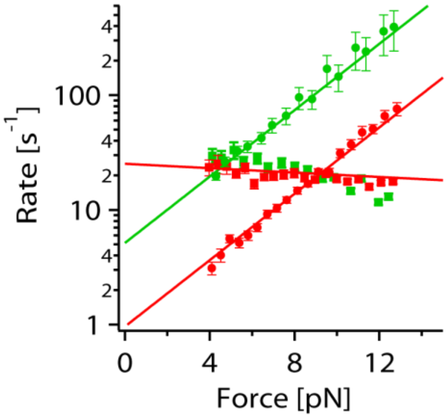

Supplementary Figure 3. Opening (circles) and closing (rectangles) rate of AdK (42-144 variant) in the presence of 100 nM AP5A and 2 mM $MgCl_2$ (red) and 100 nM AP5A and 2 mM EDTA (green).

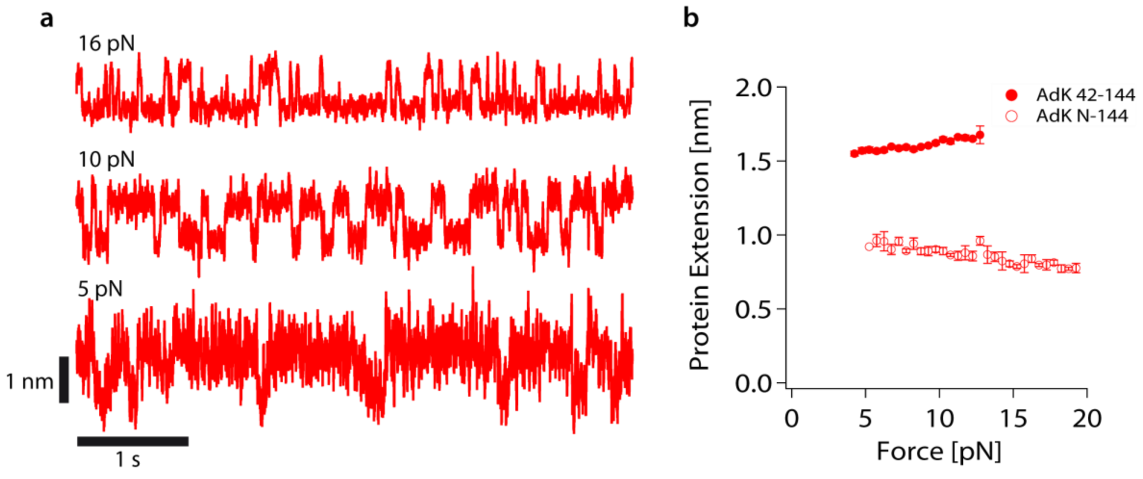

Supplementary Figure 4. (A) Sample traces of the closing and opening fluctuations of AdK pulling at the CORE domain and the ATP lid (N-144 variant) at an AP5A concentration of 30 nM AP5A and 2 mM $MgCl_2$. (B) Distance between closed and open state as a function of force in the presence of AP5A for the 42-144 variant (solid symbols) and the N-144 variant (unfilled symbols).

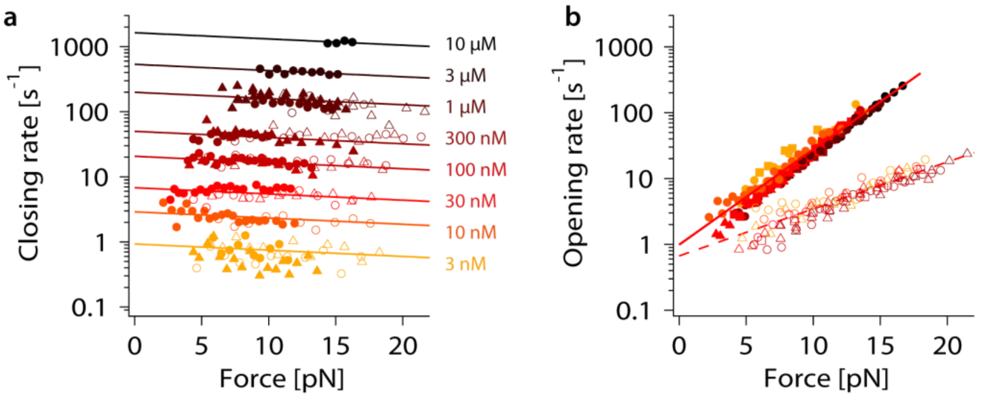

Supplementary Figure 5. Closing (A) and opening (B) rate as a function of force for different AP5A concentrations. Filled symbols represent data of the 42-144 variant and unfilled symbols of the N-144 variant. Solid lines are fits extrapolating the closing and opening rate to zero force for the 42-144 variant and the dashed line for the N-144 variant.

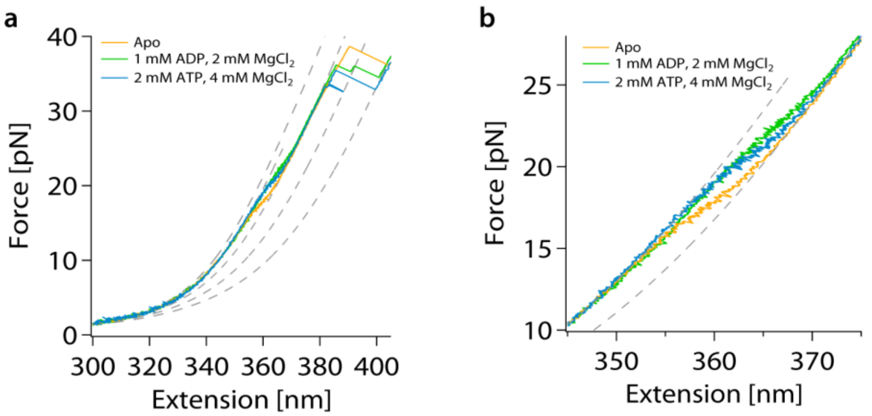

Supplementary Figure 6. Force-extension curves of AdK pulling at residue 42 and 144. The protein construct was stretched at apo conditions (orange), in presence of 1mM ADP and 2mM $MgCl_2$ (green) and in the presence of 2 mM ATP and 4 mM $MgCl_2$ (blue) at a constant velocity of 20 nm s^{-1} (low pass filtered to 60 Hz). The dashed lines correspond to WLC fits to the data. (b) shows a magnification of the unfolding/folding transition of the ATP-lid of AdK.

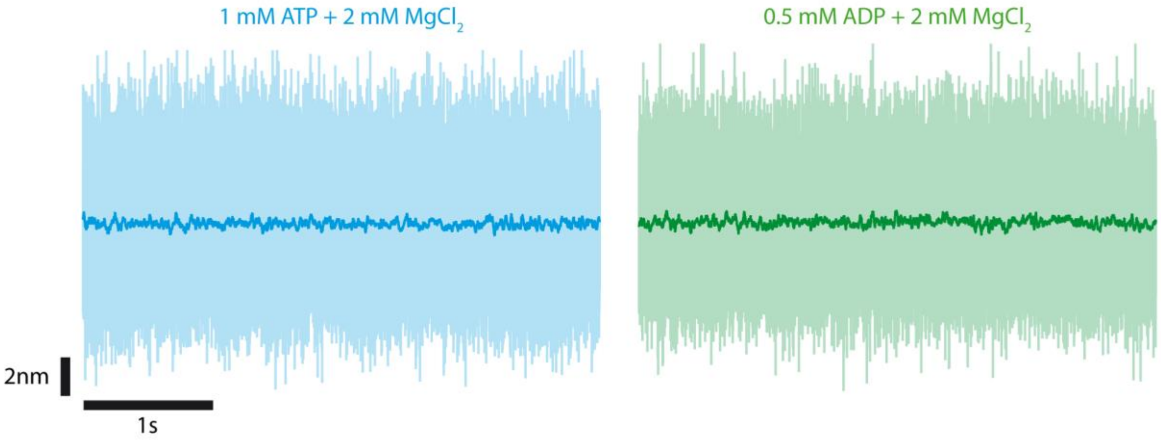

Supplementary Figure 7. Sample traces of the 42-144 variant at a force bias of 10.5 pN in presence of 0.5 mM ADP and 2 mM $MgCl_2$ (green) and 1 mM ATP and 2 mM $MgCl_2$ (blue). The light blue, respectively, light green traces show the data sampled at full bandwidth of 30 kHz and low pass filtered to 37.5 Hz in blue, respectively, green.

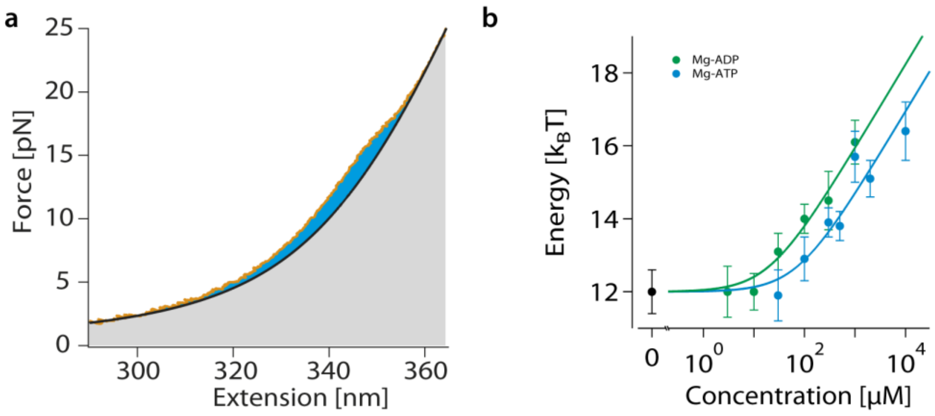

Supplementary Figure 8. (a) Force-extension curve of AdK pulling at residue 42 and 144 filtered to 60 Hz (orange). A fit of a combined WLC fit to the unfolded state is shown as a solid black line. The free energy of the folding/unfolding transition of the ATP-lid is given by the area shown in blue. The free energy of the whole system in the unfolded state is given by the area shown in gray. (b) Free energy of the fast unfolding/folding transition of the ATP lid for different nucleotide concentrations from area measurements shown in (a). Black indicates the free energy of the ATP lid in apo conditions. The fits of Equation 3 to the data yield the dissociation constants of the nucleotides to the open state of the ATP lid. The dissociation constant for Mg-ADP is (12±5) µM and for Mg-ATP (51±17) µM.

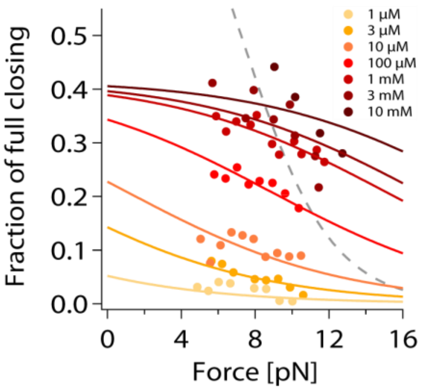

Supplementary Figure 9. ADP induced fraction of full closing as a function of force for different Mg-ADP concentrations from the AP5A competition assay. Solid lines are extrapolations of the zero-force fraction of full closing and size of the conformational change of Δx = 0.7 nm. The dashed line shows the expected force dependent closing behavior for the full closing of both lids Δx = 1.7 nm.

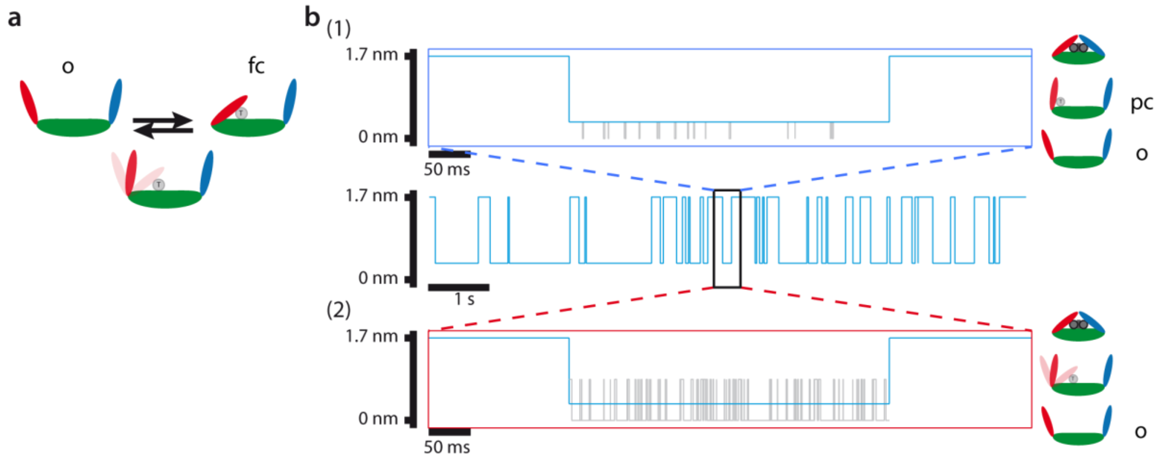

Supplementary Figure 10. Model for nucleotide induced conformational change. (A) Sketch of rapid flipping between open (left) and ATP-bound fully closed (right) state. Due to the limited time resolution, one can only observe an average between the two states (lower). (B) Middle panel shows a hypothetical trace of the observed closing and opening transitions in the AP5A competition assay with ATP (blue). The underlying fast transitions of AdK are shown as grey traces. The reduced size of the conformational change can be explained by two different models: (1) ATP drives the ATP-lid into a conformation with an only partially closed lid. (2) ATP binds to the open conformation of AdK with full affinity but only weakly drives AdK into the fully closed conformation. This leads to rapid fluctuations between the open and fully closed conformation of the ATP-lid resulting on average in an incomplete closure of the ATP-lid.

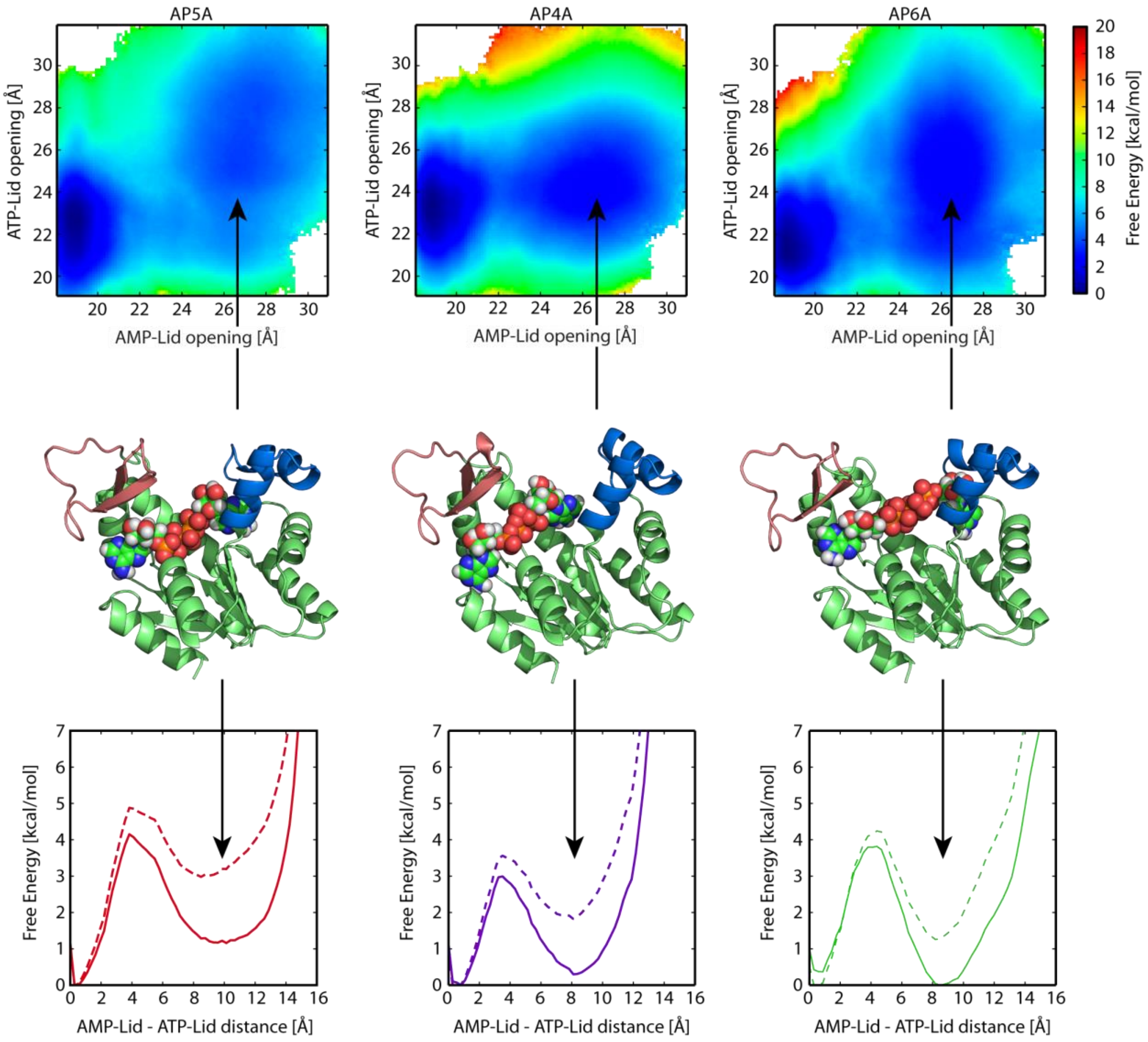

Supplementary Figure 11. Top: 2D Free Energy landscapes along AMP-lid and ATP-lid opening coordinates as obtained from 2D H-REMD-US simulations of ADK bound to the inhibitors AP5A, AP4A and AP6A. The top/left and bottom/right regions were excluded from the sampling. Middle: Exemplary snapshots [1] from the trajectories obtained in the simulations. The enzyme is depicted in cartoon representation (red: ATP-lid, blue: AMP-lid), the inhibitors as spheres. The arrows indicate the coordinate values corresponding to the shown configurations. Bottom: Projections of the 2D Free energy landscapes on a coordinate resembling the experimental lid-to-lid distance coordinate (dashed lines). To enable direct comparison with the experimental results, a bias of 15 pN was added subsequently (solid lines, see Methods).

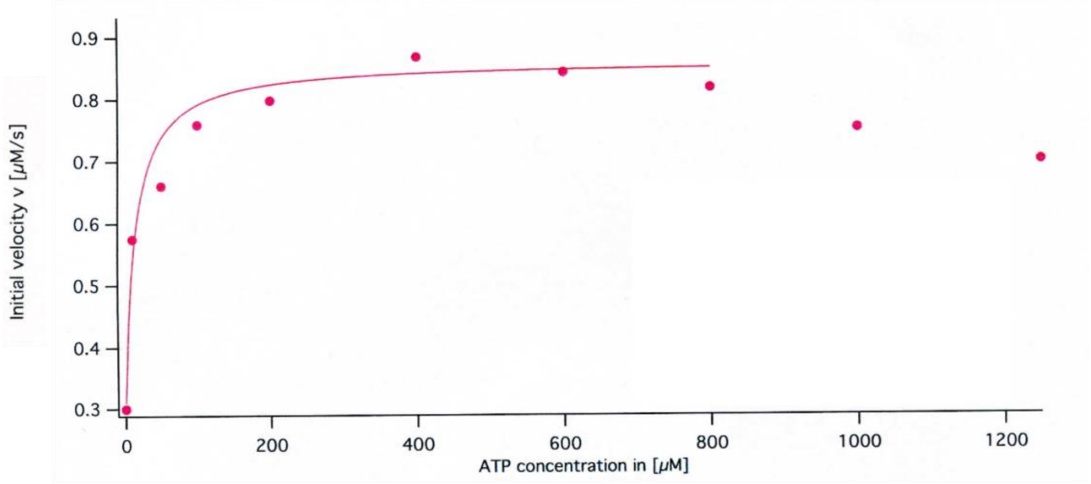

Supplementary Fig. 12. Initial velocity plot for the forward reaction (MgATP+AMP→): AdK 42-144 at 0.1 mM [AMP], 20 °C.

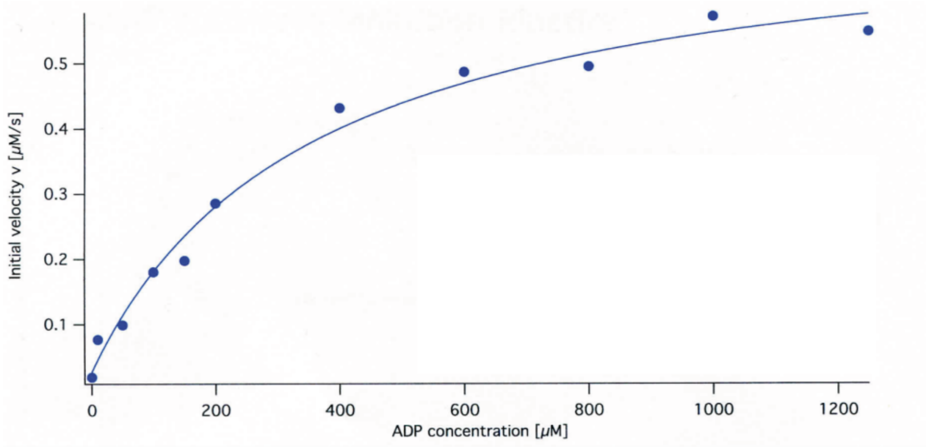

Supplementary Fig. 13. Initial velocity plot for the backward reaction (MgADP+ADP→): AdK 42-144 at 20 °C.

Supplementary References

1 *The PyMOL Molecular Graphics System, Version 1.7.4* (Schrodinger, LLC, 2010).
